# Supplementary material for: Designing a Nurse-Led Program for Self-Management of Substance Addiction Consequences: A Modified e-Delphi Study
Source: Int J Environ Res Public Health. 2023 Jan 24;20(3):2137. doi: 10.3390/ijerph20032137 (PMC9915323; doi:10.3390/ijerph20032137)
Supplement: Supplementary file 1 [file ijerph-20-02137-s001.zip › ijerph-2101606-supplementary.pdf]

Supplementary Materials -TRAINING PROGRAM FOR THE SELF-MANAGEMENT OF SUBSTANCE ADDICTION CONSEQUENCES - CONTENT

Structure

Goals:

- To empower for self-management of substance addiction consequences.
- To promote therapeutic project adherence.
- To promote Positive Mental Health.

Context of Intervention:

Setting: Location/Office that guarantees privacy.

Preferred approach: Individual, face-to-face, or virtual consultation.

Documentary support: SAC values, PMH values, Diagnoses, and Interventions.

Complementary approach: In groups of up to 4 people, whenever the follow-up processes identify benefit of "peer support and help", due to the proximity of related needs and experiences.

Nursing Process principles.

Intensity and duration:

Flexible number of planned sessions: 8

Duration of sessions: Between 20-60 min

Break between sessions: 1 x week minimum (8 weeks in total), up to 3 weeks maximum (21 weeks in total)

Inclusion criteria:

- 18 years or older.

- Included in a MAT program for at least 5 weeks.

- Followed in Nursing consultation

- Marked severity (=<48 on the Substance Addiction Consequences (SAC) Scale

- Stabilized in their psychopathological condition (if existing) to be able to interact (without changes in thought, perception, aggressiveness, or psychomotor agitation)

- No severe cognitive impairment (if necessary, using the MoCa)

Session 0
INITIAL EVALUATION
→ **Session 1**
EXPLORATION
→ **Sessions 2 to 7**
EMPOWERMENT
→ **Session 8 - FINAL SESSION**
AUTONOMY

Supplementary Materials -TRAINING PROGRAM FOR THE SELF-MANAGEMENT OF SUBSTANCE ADDICTION CONSEQUENCES - CONTENT

Content

Session 0 - INITIAL EVALUATION

- Presentation of those involved (nurse / person) - face-to-face
- Presentation of the objectives of the study and explanation of the operation of the program
- Validate inclusion criteria
- Presentation and obtaining informed consent
- Fill together the SAC scale and validate if =<48, as well as the severity in the 4 dimensions.
- Assist the person in completing the Positive Mental Health Questionnaire (PMHQ)
- Assess awareness of why "being here" evaluating readiness to change.
- Give feedback on the assessment performed (awareness of the problem (path and strategies developed), stage for change, perceived motivation), systematizing nursing diagnoses.
- Ensure the person's understanding that this is a gradual process and that the intervention will be carried out at their own pace.

Session 1 - EXPLORATION

- Welcoming the person in the consultation - face-to-face
- Stabilisation of mutual recognition of the agents involved and their role (nurse / person)
- Continue the collection of information necessary for further knowledge of the person.
- Evaluate the strategies already carried out in the past, explore the results obtained and the associated feelings
- Validate with people the nursing diagnoses chosen in the holistic assessment performed by the nurse (based on the SAC Scale, but not only, because other clinical indicators are to be considered)
- Hierarchize with the person the priority of the target foci of the intervention, and that may be simultaneously.
- Assess degree of motivation for the intervention.
- PRESCRIPTION - Stimulate reflection on the impact on your life, your problematic relationship with substances and when possible, through writing (provide paper and pen if necessary)

Beginning of all intermediate sessions

- Welcome the person in the consultation
- Discuss the requested exercise in the previous session or read with discussion (when written). Start the 2nd session with a reflection on the problematic relationship with substances in their life and current impact.
- Reassess the nursing diagnoses chosen and the path taken (feedback)
- Reassess the hierarchy of the target foci of the intervention (individualization of the time required for each person)
- Reassess degree of motivation for intervention

E M P O W E R M E N T

**Diagnostics / Interventions – Related to**
**Concerning the problematic use of substances**
**Problematic use of substances**
**Health knowledge in general**
**Health search behavior and support**
**Self-knowledge and well-being**
**Social role and personal dignity**

End of all intermediate sessions

- Perform the summary and evaluation of the session
- Negotiate the objectives to be achieved and the tasks to be performed until the next session, guided by hierarchy.
- Motivate for continuity in the program

Final Session (8) - AUTONOMY

- Reassess the status of all nursing diagnoses elected - face-to-face.
- Fill together the SAC scale and validate whether > 48
- Assist the person in completing the PMHQ
- Assess the degree of motivation to pursue self-management strategies for their problem
- Strengthen strategies to meet the challenges that the person can encounter in his/her daily life.
- To consider the continuity of the follow-up process by the nursing team, by maintaining significant severity (SAC =<48) or for not improving the status of diagnoses.
- Schedule follow up.
